# Discharge service as a determinant of 30-day readmission in a cohort of maintenance hemodialysis patients: a retrospective cohort study

**DOI:** 10.1186/s12882-017-0761-9

**Published:** 2017-12-04

**Authors:** Ladan Golestaneh, Eran Bellin, William Southern, Michal L. Melamed

**Affiliations:** 1 Department of Medicine/ Renal Division, Montefiore Medical Center/Albert Einstein College of Medicine, 3411 Wayne Ave, Suite 5H, Bronx, NY 10467 USA; 2 Department of Epidemiology and Population Health, Montefiore Medical Center/Albert Einstein College of Medicine, Bronx, USA; 3 Division of Hospitalist Medicine, Montefiore Medical Center, Bronx, USA

**Keywords:** Dialysis hospitalization teaching service

## Abstract

**Background:**

End stage renal disease (ESRD) patients on maintenance hemodialysis, are high utilizers of inpatient services. Because of data showing improved outcomes in medical patients admitted to hospitalist-run, non-teaching services, we hypothesized that discharge from a hospitalist-run, non-teaching service is associated with lower risk of 30-day re-hospitalization in a cohort of patients on hemodialysis.

**Methods:**

One thousand and 84 consecutive patients with ESRD on maintenance hemodialysis who were admitted to Montefiore, a tertiary care center, in 2014 were analyzed using the electronic medical records. We evaluated factors associated with 30-day readmission in multivariable regression models. We then tested the association of care by a hospitalist-run, non-teaching service with 30-day readmission in a propensity score matched analysis.

**Results:**

Patients cared for on the hospitalist-run, non-teaching service had lower socio-economic scores (SES) and had longer lengths of stay (LOS), as compared to a standard teaching service, but otherwise the populations were similar. In multivariable testing, severity of illness, (OR 2.40, (95%CI: 1.43–4.03) for highest quartile) number of previous hospitalizations (OR 1.22 (95%CI:1.16–1.28) for each admission), and discharge to a skilled nursing facility (SNF)(OR 1.56 (95%CI:1.01–2.43) were significantly associated with 30-day re-admissions. Care by the non-teaching service was associated with a lower risk of 30-day readmission, even after adjusting for clinical factors and matching based on propensity score (OR 0.65(95%CI:0.46–0.91) and 0.71(95%CI:0.66–0.77) respectively).

**Conclusions:**

Patients with ESRD on hemodialysis discharged from a hospitalist-run, non-teaching medicine service had lower odds of readmission as compared to those patients discharged from a standard teaching service.

## Background

Patients on maintenance hemodialysis represent the highest health care expenditures by diagnosis. Patients with ESRD make up 1% of the Medicare beneficiary population but account for 8% of Medicare payments and spend, on average, 14 days per year in the hospital [1]. Thirty to 35% are re-hospitalized within 30 days of discharge compared to 18% of the general Medicare population [1–4]. In response to this crisis, the Centers for Medicare Services (CMS) has made 30-day hospital readmission a dialysis facility quality metric [5]. Patients on hemodialysis are high utilizers of inpatient services because of extremely complex clinical needs in addition to psychosocial needs that are amplified in the face of demanding treatment schedules and restrictive lifestyle requirements [1, 3, 6–9]. While hospitalized, these patients experience a decline in their nutritional status and mobility, and inconsistencies in their dialysis prescription and medication regimens [9, 10]. The high burden of inpatient hospitalizations in dialysis patients not only signifies complex comorbidities and shortfalls in outpatient care delivery, but directly contributes to high morbidity and mortality [2, 9, 11].

Studies show that risk factors for frequent hospitalizations in hemodialysis patients include inadequate transitions and discharge plans between institutions [2–4, 12–15]. Discharge planning plays a crucial role in the post-discharge outpatient coordination since dialysis patients have the added layer of complexity of the dialysis center schedule [2, 4, 11, 16–18]. Furthermore, the lack of a common communication portal between outpatient dialysis centers, hospital discharge teams and outpatient clinics make coordination difficult [5, 17–20]. In a recent study by Harel et al. the number of complicated care transitions was significantly associated with a higher risk of hospital readmissions in a population of Canadian ESRD patients [17]. Hall et al. describe a 30% rate of hospitalization or ED visit within 30 days after discharge from a skilled nursing facility in a cohort of 1223 prevalent ESRD patients [18]. It is remarkable that this high hospitalization rate occurs even though outpatients receiving hemodialysis have at least 3 interactions per week with healthcare staff [8, 18]. These findings highlight the institutional and clinical complexities of ESRD care. Discharge planning and discharge instructions have been evaluated as interventions in hospitalized patient populations and those interventions that support self-care and include short term follow-up, are effective in reducing hospital readmissions [21, 22].

To address the need for interventions that effectively reduce re-hospitalization rates in the maintenance hemodialysis population we aimed to refine our understanding of aspects of the index hospitalization that associate with within 30-days readmission rates. Studies have shown that hospitalist run, non-teaching medical services are associated with reduced lengths of stay, reduced readmission rates and provide efficient, high quality inpatient services to medical patients in academic medical centers [19, 20, 23, 24]. A single-center study and a systematic review, however, found no difference in mortality or 30-day readmission rates between general internal medicine patients admitted to teaching as compared to those admitted to non-teaching services [25, 26]. The exact composition of service teams in the studies cited above are not known and are likely inconsistent. No studies have examined the performance of a hospitalist-run, non-teaching, service in the care of hospitalized patients on hemodialysis, a group of patients with complex hospitalization and discharge needs.

In our institution, the hospitalist-run, non-teaching medical services utilize physician assistants who round with the attending hospitalist. Bed assignments to the hospitalist non-teaching vs house-staff run teaching services are done by administrators in the order in which beds are requested without consideration of admitting diagnosis or patient complexity. We aimed to compare the readmission rates for hemodialysis patients cared for by hospitalist-run, non-teaching versus standard teaching care teams in a large urban tertiary-care medical center. We further aimed to examine risk factors for 30-day readmission in the same cohort of hemodialysis patients in order to understand the role of discharge service, in the context of other risk factors for hospital readmission.

## Methods

### Study setting

**Fig. 1:**
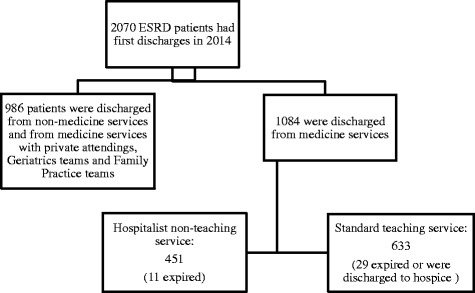
Flow-chart of study population and their outcomes

The Montefiore Health System is a large urban academic hospital system that serves the Bronx and is unique in its close proximity to a large number of dialysis centers. We used Montefiore Medical Center’s clinical database, Clinical Looking Glass™, to build a cohort of initial patient discharges in 2014 from any of the main hospitals in the Montefiore system with the diagnosis code of ESRD (*n* = 2070) (ICD9 = 585.6 as ICD10 coding was not in use in our facility in 2014) (Fig. 1). Looking GlassTM Clinical Analytics (Streamline Health, Atlanta, Georgia) is a user-friendly interactive software application for the evaluation of health care quality, effectiveness, and efficiency. The system integrates clinical and administrative datasets allowing non-statisticians to produce epidemiologically cogent self-documenting reports globally assessing care quality while identifying the specific patients in need of clinical remediation. Transplant recipients were automatically excluded because in our institution they are admitted to the surgical transplant services and not Medicine services. Furthermore, at the time of the study less than 6% of patients were on peritoneal dialysis, and were not included in the cohort. Using the Clinical Looking Glass database we examined clinical characteristics, previous hospitalizations, and readmission to any hospital in the Montefiore Health System. The Institutional Review Board at Montefiore Health System approved the study protocol and we adhered to the principles of the Declaration of Helsinki.

### Study cohorts

To test our hypothesis, we included only those subjects discharged from medicine services and compared care by hospitalist-run, non-teaching service to care by the standard teaching service. The standard medicine teaching services in our institution is supervised by hospitalists, general internal medicine and sub-specialist attendings. We excluded those subjects discharged from private attending-run, geriatric or family practice medicine services. For the propensity score models we included those patients discharged from a hospitalist-run, non-teaching medicine service who had a propensity score match to a patient discharged from a standard teaching medicine service and who had not died or been discharged to hospice (see below, under “statistical analysis”).

### Study variables

Demographic variables were collected including age, gender, race (was self-reported in the Montefiore Health System EMR and was categorized into “White”, “Black”, “Hispanic” and “Other”). The following data were also extracted: discharge service, Diagnosis Related Code(DRG) weight, (which was developed as a metric of illness severity based on 3 M coding rules and measures resource utilization for an inpatient episode as a proxy for level of acuity and care intensity) [27], insurance coverage at index hospitalization (Commercial, Medicare, Medicaid), Charlson comorbidity score, including history of heart disease, malignancy and diabetes, and discharge diagnosis of index hospitalization (based on ICD-9 coding). DRG weights were broken into quartiles and labeled from least severe to most severe. The diagnostic categories derived for this study combined ICD coded diagnoses and categorized them based on ESRD relevant diagnoses. (eg. heart failure and hypertension was recoded as fluid overload). This recoding was performed by an author (LG) based on a review of the literature pertaining to studies of this type and the classification was validated by 3 other blinded nephrologists who were unassociated with the study [11, 18]. Table 1 describes the classification used to categorize the 6 groups. Mean socio-economic status was based on attributes from census track and census block creating a smaller unit of geographic areas which then corresponds to a state mean adjusted score, with negative scores representing values below New York State’s mean scores [28]. Each score includes consideration of income, value of housing units, education, and occupation. The median New York State family income is $60,850 per year [29]. Each integer value corresponds to one standard deviation away from the mean score for New York State [28]. Hospitalist-run non-teaching service versus standard teaching service was assigned based on a service code assigned at the time of discharge. Hospitalist-run non-teaching services included a physician assistant (PA) and a hospitalist, as compared to standard teaching service which included a house-staff team in lieu of a PA and a teaching attending. Patients cared for by private (voluntary) physicians were excluded. The length of stay (LOS) was also obtained as was disposition from the index hospitalization which was re-categorized into six categories per location of discharge and services rendered: home, homecare, SNF, discharge “against medical advice (AMA)”, hospice and death during index hospitalization. The first serum albumin level from the index hospitalization was reported, as were the number of (count) prior admissions to the Montefiore Health System over a 365 day period.

**Table 1:** Diagnostic Category Derivation

Assigned Diagnostic Category	ICD-9 defined diagnosis
Cardiac	Myocarditis, ischemic cardiomyopathy, MI, non-specific chest pain
Fluid related	Congestive heart failure, fluid and electrolyte disorder, syncope
Access related	Septicemia, complication of device, dialysis access malfunction
Pulmonary	Asthma, Pneumonia, Chronic obstructive pulmonary disease and bronchiectasis
Anemia	Gastrointestinal hemorrhage, deficiency and other anemia
Other	All others

*ICD* International Classification of Diseases

### Outcome variable

The primary outcome variable was readmission to the Montefiore Health System within 30 days of discharge from the index hospitalization.

### Statistical analysis

We first evaluated differences between patients cared for by the hospitalist-run, non-teaching versus the teaching services using t-tests, the Mann-Whitney test or chi-square test depending on the distribution of the variables. To examine the association between care by the hospitalist-run, non-teaching service and readmission we built univariate and multivariable logistic regression models. First, in an unadjusted analysis, we constructed a univariate logistic regression model with 30-days readmission as the outcome, and discharge service code (hospitalist-run, non-teaching versus standard teaching) as the single independent variable. Next, we constructed a multivariable model, adjusting for demographic characteristics of patients only. Finally, to examine the independent associations between discharge service and readmission we constructed a multivariable model using stepwise backward elimination (threshold *p*-value 0.1) with the outcome variable of 30- days’ readmission and the independent variable of interest of discharge service. Since length of stay and disposition to SNF were different between the two discharge services groups, we ran an additional analysis including the two variables, individually, in the final model to evaluate for any residual confounding and mediation effects. Post estimation testing utilized Hosmer-Lemeschow statistic in addition to AUC curves to assess the predictive capability of the logistic regression model. In addition to evaluating whether service type was associated with 30- day readmissions, we evaluated other variables associated with the outcome using multivariable logistic regression as above. We conducted sensitivity analyses to test the association between within 3 days readmission, as a potential indicator of “avoidable” re-admissions and discharge service in order to test the association of discharge service with potentially avoidable readmissions. In keeping with our intent, we also conducted a sensitivity analysis that tested the ICD-9 category of readmission and its association with index discharge service using Chi-Square Analysis.

In a secondary analysis to test the robustness of the associations, we also calculated the risk of 30-day readmission in a propensity-matched sub cohort. Propensity score matching is an established method to address a major limitation of observational studies, namely, confounding by indication [30, 31]. A propensity score is calculated as the estimated probability from logistic regression of a patient’s being assigned to a given intervention, in this case, the hospital service. The following variables were used in the regression model to derive this propensity score for each patient: age, gender, race, albumin level, Charlson comorbidity score, history of severe heart disease, and history of malignancy, and number of previous admissions within 1 year, standardized SES, and DRG derived index severity of illness quartile. We did not match based on disposition and LOS because they were not known at the time of patients’ hospitalization, but we reported them. We did conduct sensitivity analyses to test the confounding and modifier effects of index LOS on the association between discharge service and odds of 30 days readmission. LOS was examined as a continuous variable and in tertiles. Variables were chosen based on a non-parsimonious approach. We then matched the cohort based on propensity score blocks using nearest neighbor one-to-one matching without replacement, and tested the association of discharge service with 30-day readmission in those patients in the matched cohort. In a sensitivity analysis, we evaluated the inverse probability propensity score models and found no difference from the propensity matched model. Two sided *p*-values of 0.05 or less were considered significant.

## Results

### Baseline characteristics of the total cohort

Out of a total of 1084 subjects discharged from a medical service in 2014 with a diagnosis of ESRD and on maintenance hemodialysis, 284 or 19.8% were readmitted within 30 days to the Montefiore system while 79 or 5.4% expired or were discharged to hospice (Fig. 1 and Table 2). Overall, the mean age of all patients was 61.3 years, and 48.5% were non-Hispanic Black, 8.1% were non-Hispanic White and 23.2% identified as Hispanic. The preferred language for 19.8% of the patients was Spanish, and 7.5% had commercial insurance. Forty -2 % of all patients were discharged from the hospitalist-run, non-teaching medicine service, 45.1% of patients were discharged home, 21.9% were discharged to SNF and 24.4% were discharged home with home-care. Fifty four percent of the discharged population had had at least one previous hospitalization in the previous year with 22% having one hospitalization and 32% of the total cohort having two or more hospitalizations. The median SES was −2.80 (25–75% IQR: -6.40-(−1.10)) standardized to the New York State mean income, the median length of stay was 6 days (25–75%IQR:3–12), the mean Charlson score was 5.2 (SD: 2.3) and the mean albumin level was 3.75 mg/dL (SD: 0.6).

**Table 2:** Common variables and their associations with discharge service in the total cohort and in the propensity matched cohort

	Patients on Hospitalist run Non-Teaching Service (*n* = 451)	Patients on Standard Teaching Service (*n* = 633)	*p*-value	Propensity matched patients on Hospitalist Service (*N* = 425)	Propensity matched patients on Teaching Service (*N* = 425)	*p*-value
Age (years): (*n* = 1084)						
Mean (SD)	61.1(14.8)	61.4(14.1)	0.72	61.6	61.4	0.89
Gender: (*n* = 1084) *n*(%)						
Male	215(47.7)	306(48.3)	0.85	198(46.6)	201(47.3)	0.83
Female	236(52.3)	327(51.7)		227(53.4)	224(52.7)	
Race: (*n* = 1084) *n*(%)						
Non Hispanic Black	224(49.7)	322(50.9)		209(49.2)	218(51.3)	
Non-Hispanic White	21(4.7)	40(6.3)		21(5.0)	28(6.6)	
Hispanic	116(25.7)	142(22.4)	0.36	113(26.6)	88(20.7)	0.75
Multiracial	5(1.1)	14(2.2)		4(0.9)	10(2.3)	
Other	85(18.8)	115(18.2)		78(18.3)	81(19.1)	
Severity of Illness (by quartile)(*N* = 1084) *n*(%)						
Extreme	58(12.9)	106(16.8)		55(12.9)	65(15.2)	
Major	111(24.6)	146(23.1)	0.21	106(24.9)	89(20.9)	1.0
Moderate	136(30.2)	201(31.8)		128(30.1)	132(31.1)	
Minor	146(32.4)	180(28.4)		136(32.1)	139(32.8)	
SES (*n* = 1065 (median (IQR))						
(with 0 as NYS mean)	−3.47(−6.45-(−1.55))	02.80(−6.32-(−1.10))	0.003	−3.98	−3.92	0.76
Albumin (mg/dL) (*n* = 1036) mean (SD)^a^	3.75(0.03)	3.73(0.03)	0.61	3.74	3.74	0.98
History of Severe Heart Disease (*n* = 1084)(%)	27(6.0)	69(10.9)	0.005	26(6.2)	24(5.6)	0.77
History of Malignancy (*n* = 1084) (%)	7(1.1)	7(1.6)	0.59	6(1.5)	5(1.1)	0.52
Charlson score: (*n* = 1084) median (IQR))	5(3–6)	5(4–6)	0.19	5.2	5.2	0.89
Number of previous admission within 365 days (*n* = 1084)(median (IQR))	1(0–2)	1(0–2)	0.71	1.95	1.73	0.28
Outcome variables not used in matching						
LOS (days) in index hospitalization (*n* = 1084) Days (median (IQR))	6(3–13)	5(3–10)	0.014	12.1	9.5	0.02
Disposition from index hospitalization (*n* = 1060) *N* (%)						
Home (no services)	228(51.5)	281(45.5)		207(48.7)	191(44.9)	
Skilled Nursing	62(14.0)	131(21.2)	0.004	61(14.3)	84(19.8)	0.52
Home care	127(28.7)	154(25.0)		126(29.6)	108(25.4)	
AMA^b^	15(3.4)	22(3.6)		20(4.8)	25(5.9)	
Expired/Hospice	11(2.5)	29(4.7)		11(2.6)	2(4.0)	

*SES*: Socio-economic status; *LOS*: length of stay

^a^Only variable means displayed for the matched samples

^b^ *AMA* Against medical advice

### Characteristics of the total cohort and propensity-matched cohort: Hospitalist-run, non-teaching versus standard teaching service

**Fig. 2:**
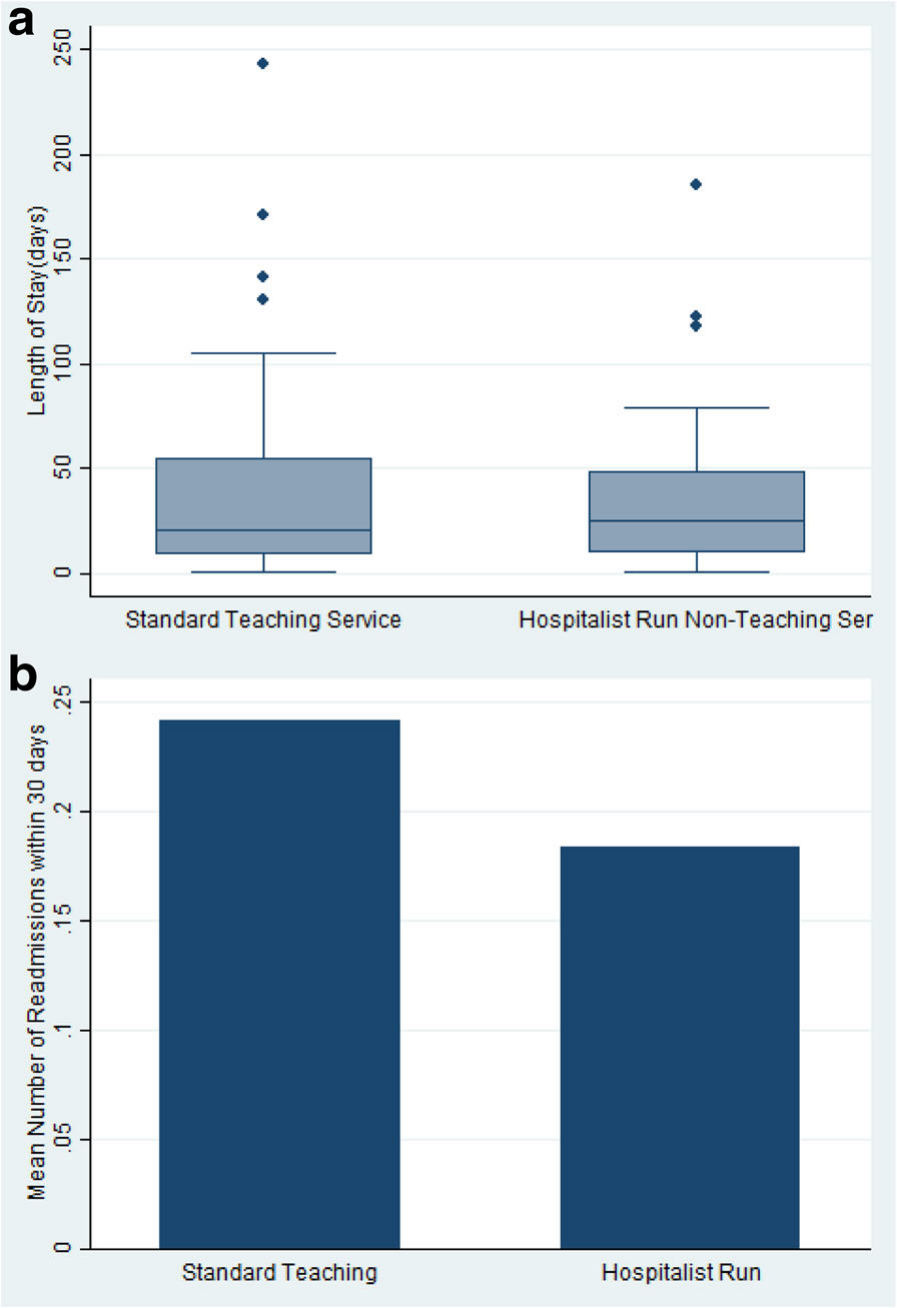
Characteristics of the Services. **a**: Length of Stay in Each Service; **b**: Rate of Readmission within 30 days by Service Type

We compared the association of each variable with discharge service (hospitalist-run, non-teaching versus teaching) in the total cohort and found the mean SES to be lower among patients cared for by the hospitalist-run, non-teaching service (−3.47 as compared to −2.80) in the teaching service (Table 2). All other variables, including age, gender, race, Charlson score, DRG based severity of illness quartile, albumin level and number of previous admissions was not associated with discharge service (Table 2). Disposition and hospital LOS remained significantly different in those patients matched based on propensity scores (Table 2, Fig. 2a). Otherwise, the propensity matched cohorts were well balanced and showed similar associations with all other variables (Table 2).

### Associations between hospitalist-run, non-teaching versus standard teaching service and 30-day readmissions

Discharge from a hospitalist-run, non-teaching medicine service was associated with a lower odds of 30-days readmission (odds ratio (OR) 0.68; 95% confidence intervals (CI): 0.50–0.92) (Table 3). The protective association between hospitalist-run, non-teaching care and reduced risk of 30-day readmission remained strong and significant after adjustment for demographic characteristics (OR 0.68; 95% CI: 0.51–0.93) and in full multivariable model adjusting for clinical characteristics (OR 0.65; 95% CI: 0.46–0.91) (Table 3). The C-statistic for the final model was 0.722. Of the 431 patients cared for on the hospitalist service in the full cohort, 425 were matched to 425 patients cared for on the teaching service. The matched cohorts were similar with respect to demographic and clinical characteristics (Table 2). In the matched cohorts, care by the hospitalist service was associated with significantly reduced odds for 30-day readmission (OR = 0.71; 95% CI: 0.66–0.77) (Table 3). Sensitivity analyses showed a seeming protective effect of hospitalist-run non-teaching service in up to 3 day readmissions as compared to standard teaching service. However, because of the few number of events (33) within this time period, there was not enough power to show a statistically significant difference between discharge services with reference to short term readmissions. In addition, sensitivity analysis did not show a confounding or interaction effect of index LOS on the association between index discharge service and odds of 30 day readmission.

**Table 3:** Associations between medical service and 30-day readmission in patients on mainteinance hemodialysis

	OR (95% CI)	*p*-value
Unadjusted	0.68(0.50–0.92)	0.014
Model 1 (demographics)^a^	0.68(0.51–0.93)	0.014
Model 2 (fully adjusted model)^b^	0.65(0.46–0.91)	0.012
Propensity matched cohort	0.71(0.66–0.77)	0.009

^a^included age, gender, and race variables

^b^included age, gender, race, diagnosis related group(DRG) weight quartile, albumin, Charlson severity score,number of previous admissions, socio-economic status (SES)

### Common variables associated with 30-day readmission: Univariate and multivariate testing

There were no differences in mean age, gender distribution or race category between the group that was readmitted and the group that was not (*p* = 0.35, 0.32, 0.25, respectively)(Table 4). Discharge from a non-teaching service was significantly protective against readmission within 30 days (OR:0.68(95% CI: 0.50–0.92) (Table 4, Fig. 2b). Those patients that were readmitted within 30 days had a median of 2 (25–75% IQR):1–4) hospital admissions during the previous year, as compared to a median of 0 (25–75% IQR:0–2) previous admissions in those patients that were not readmitted. Those readmitted within 30 days of discharge had higher DRG weighted illness scores and higher LOS. (6 days (IQR: 3–13 vs. 5 days (IQR: 3–10) respectively) (Table 4) (*p* = 0.03). Notably, those patients that had a 30-day readmission were more commonly discharged to SNF and home with home-care services after the index hospitalizations (23.6% vs 17.6% respectively for SNF, 33.2% vs 25.9% respectively for homecare; Table 4). In our multivariate regression model, 960 subjects had all variable data points and did not expire or get discharged to a hospice facility. The highest quartile for the severity of illness weight had a 2.40 times odds of 30-day readmission as compared to the lowest quartile (95% CI: 1.43–4.03) (Table 4). Those patients discharged to a SNF from the index hospitalization had 1.56 odds of 30-day readmission (95%CI, 1.01–2.43) (Table 4). Finally, for every episode of prior hospitalization within 365 days, there was a 1.22 odds of 30 day future readmission (CI: (1.16–1.28) (Table 4). Inclusion of LOS and discharge to SNF variables to the final model did not significantly alter the odds of readmission for the hospitalist-run, non-teaching service cohort as compared to the standard teaching service.

**Table 4:** Logistic regression model with 30 days readmission as outcome variable (excluding those patients that died in the hospital or were discharged to hospice from the index hospitalization)

	Subjects readmitted within 30 days	Subjects not re-admitted within 30 days	*P* Value	Odds of re-admission within 30 days (95% CI)(*N* = 960)
Age (*N* = 1084) (mean, SD)	61.4(14.4)	60.4(15.1)	0.35	0.99(0.98–1.00)
Male gender (*n *(%))	106(44.9)	396(48.6)	0.32	0.89(0.65–1.23)
Race (1020) (*n*, %)				
Non-hispanic White	16(6.8)	45(5.3)		1
Non-hispanic Black	105(44.5)	441(52.0)	0.25	0.92(0.46–1.85)
Hispanic	65(27.5)	193(22.8)		1.28(0.62–2.65)
Multiracial	3(1.3)	16(1.9)		1.08(0.24–4.78)
Other	47(19.9)	148(18.0)		1.25(0.59–2.64)
Severity of illness (*n*(%))				
Minor	55(23.3)	271(32.0)		1
Moderate	70(29.7)	267(31.5)	0.01	2.24(0.80–1.91)
Major	63(27.7)	194(22.9)		1.61(1.02–2.57)
Extreme	48(20.3)	116(13.7)		2.40(1.43–4.03)
Disposition				
Home	89(38.9)	420(53.1)		1
Skill Nursing Facility	54(23.6)	139(17.6)	0.002	1.56(1.01–2.43)
Homecare	76(33.2)	205(25.9)		1.44(0.98–2.11)
AMA	10(4.4)	27(3.4)		1.88(0.83–4.22)
Prior admission in the past 12 months (median, IQR)	2(1–4)	0(0–2)	<0.001	1.22(1.16–1.28)
Albumin (mg/dL)	3.71(0.6)	3.76(0.6)	0.35	0.82(0.62–1.08)
Charlson Score (mean, SD)	5.5(2.4)	5.2(2.4)	0.08	0.97(0.91–1.05)
SES (median, IQR)	−3.03(−5.9-(−1.2))	−3.04(−6.4-)-1.2))	0.90	1.00(0.94–1.06)
Non-teaching Service	368(43.4)	83(35.2)	0.02	0.65(0.47–0.91)
Teaching Service	480(56.6)	153(64.8)		1

*SES* Socio-economic status

## Discussion

Our study of a cohort of hospitalized patients in maintenance hemodialysis discharged from the Montefiore Health System in 2014 showed that discharge from a hospitalist-run, non-teaching service was significantly associated with a lower risk of 30-days readmission. This relationship remained significant after multivariable adjustment and use of propensity score matched cohorts. Our data also showed illness severity during the index hospitalization, previous number of hospitalizations and discharge to SNF as significantly associated with 30-days readmissions. To our knowledge, this is the first analysis of hospital service as a predictor of 30-day readmission in patients on maintenance hemodialysis.

Interestingly, hospitalist-run, non-teaching services had a significantly higher LOS as compared to standard teaching services, and a lower rate of discharge to SNF. Perhaps the longer LOS was instrumental for safe discharge planning in this population, though to explore the role of LOS as a mediator of readmission risk, we ran analyses with discharge to LOS as a variable in the model. Inclusion of LOS in the model did not alter the OR for readmission according to discharge service. Of note, those patients admitted to the standard-teaching service had a higher incidence of death and discharge to SNF. (3.6% vs. 2.5%; 21.2% versus 14.0%, respectively). These differences, though small, could be accounted for by residual confounding. However, if patients discharged from the teaching service was more likely to die (because they were more severely ill, for example) then their risk for readmission would be lowered, unlike what we showed. Discharge to SNF, however, is worth exploring in future studies, as a source of differential bias in our analysis.

In the wake of the Affordable Care Act, CMS launched a “hospital readmission reduction program” wherein hospitals are financially penalized for frequent readmissions [32]. Though their proposed financial penalties apply to a few admission diagnoses, there is a clear move to increased scrutiny of all short-term readmissions, including those occurring in patients on maintenance hemodialysis. First dialysis occurring in hospital (hazard ratios (HR) 2.1, 95% CI 1.4–3.3, *P* = 0.0005), the use of a central venous catheter at first hemodialysis (HR 2.6, CI 1.6–4.4, *P* < 0.0001), hypo-albuminemia, and cognitive and physical decline are a few known predictors of 30 days readmission [2, 10, 17, 33]. We identified discharge to SNF, higher severity of illness scores, higher number of previous hospitalizations and hospital service as significantly associated with 30-days readmissions. Though patients discharged to SNFs suffer from higher burdens of illness, our multivariable model adjusted for comorbidity scores and severity of illness. This observation is likely related to the very specialized care these patients require, wherein SNFs may not be able to handle these clinical complexities which can result in a reflexive referral back to the hospital. Approximately 20–25% of discharges to nursing homes are readmitted within 30 days in a general medical population and the on average, nursing home residents are sent to ED twice per year with potentially avoidable reasons (with normal vital signs and no diagnostic tests). [34, 35]Furthermore, Pia et al. showed that because of the higher number of transitions involved in discharging patients with ESRD, an added layer of complexity involving SNF and nursing home hand-offs are detrimental and increase the risk of re-hospitalization [8].

A comparison of clinical outcomes in teaching and non-teaching general internal medicine services shows equivocal results. In academic hospitals, admission to a medicine unit run by a teaching service showed similar costs and clinical outcomes as compared to those admitted to a non-teaching service [26]. Discharge from teaching hospitals of patients with severe heart failure exhibited better outcomes as compared to discharge from non-teaching hospitals [36]. A recent meta-analysis of all studies examining outcome differences between teaching versus non-teaching healthcare structures, failed to show a difference overall [37]. The hemodialysis population has more complex care transitions than other types of populations, and effective coordination and implementation of discharge planning is crucial to readmission outcomes. Previous studies have identified poor post-discharge planning, complex institutional transitions and care coordination as process factors responsible for short term readmissions in this population [2, 5, 18, 38]. These studies posited that complex discharge planning between episodes of care and over-surveillance of clinical parameters in SNF and hospital settings contribute to referrals made to inpatient settings [3, 32, 38]. Our results suggest that non-teaching hospitalist services may be more adept at discharge planning and complex institutional transitions.

The process measures responsible for our findings of the protective effect of non-teaching service on readmission may include timely interaction with patients, immediate availability during working hours and acquired expertise with coordinating inpatient provisions and discharge planning [24, 39, 40]. In our institution, the hospitalist-run, non-teaching service has immediate access to patients upon their admission, and with the help of the PA, the hospitalist attending takes a hands-on approach to management. Our data suggest that it may be this hands-on approach of hospitalist attendings experienced with the care of hemodialysis patients, and sometimes with the recurrent care of the same hemodialysis patients, that makes them more adept at connecting with outpatient dialysis centers and nephrologists to synthesize more effective discharge plans. Furthermore, the structure of hospitalist/PA run service rounds including daily and early interactions between attendings and social workers may result in more effective discharge plans. No studies have compared readmission risk differences between teaching and non-teaching services in the ESRD population. Our finding presents an opportunity for researchers and policy makers to identify aspects of a medicine hospitalist service that associate with protection against readmission. Further studies are needed to explore the elements of non-teaching service including effective discharge planning and their association with readmission risk.

Limitations of our study relate to its observational design which cannot ascertain causality and the fact that it examines one medical system which limits generalizability. However ESRD is highly prevalent in inner city minority populations like ours. We also lack accurate outpatient death data for the 30 days post-admission. However, sensitivity analysis showed that up to 75% of the original cohort had readmissions within 1 year to Montefiore which suggests, at the very least, that the majority were accounted for up to 1 year after index admission and had not died. Our SES data was based on census data and individual data. Furthermore, we eliminated expired patients and those patients discharged to hospice from the analysis. Our sensitivity analysis was not powered to show a statistically significant difference between discharge services with reference to within 3 days readmission, and time to readmission should be explored further as a variable in future research examining mediators of readmissions in this population. There is a possibility that patients were readmitted to other hospitals in the area with resultant under-reporting of 30-day re-hospitalization and/or selection bias, however Montefiore Health System is the largest hospital system in the Bronx and it covers multiple neighborhoods that span the borough. There is no reason to believe that there would be a differential bias between discharge services and readmissions to outside hospitals. Other potential confounders for which we could not account include lack of data regarding the location from which the patients were admitted and lack of data regarding ICU stay of the patients during index hospitalization. Residual confounding, the inability to adjust for variable or elements not included in the analysis is also playing a role. Our study also had several strengths including a large sample size, detailed clinical, laboratory and demographic information, a diverse patient population and the consistency in patterns of care offered by a single center. Furthermore, the variables included in our model had previously been shown to impart high discriminative ability in readmission risk scores [32, 38].

## Conclusions

In conclusion, our study supports the hypothesis that assignment to a hospitalist-run, non-teaching medical service is associated with fewer 30-day re-hospitalizations in a cohort of ESRD patients. The qualities unique to a non-teaching service include earlier triage of patients by attending, longer LOS, and early interactions with social workers and care coordinators are worth exploring to help identify factors that are protective against frequent readmissions.

## Abbreviations

AMA: Against medical advice
AUC: Area under the curve
CI: Confidence interval
CMS: Centers for Medicare Services
DRG: Diagnosis related group
ESRD: End Stage Renal Disease
HR: Hazard ratio
IQR: 25–75% inter-quartile range
LOS: Length of stay
OR: Odd ratio
SD: Standard deviation
SES: Socio-economic status
SNF: Skilled nursing facility
